# Influenza A Virus PA Antagonizes Interferon-β by Interacting with Interferon Regulatory Factor 3

**DOI:** 10.3389/fimmu.2017.01051

**Published:** 2017-09-11

**Authors:** Chenyang Yi, Zongzheng Zhao, Shengyu Wang, Xin Sun, Dan Zhang, Xiaomei Sun, Anding Zhang, Meilin Jin

**Affiliations:** ^1^State Key Laboratory of Agricultural Microbiology, Huazhong Agricultural University, Wuhan, China; ^2^Laboratory of Animal Virology, College of Veterinary Medicine, Huazhong Agricultural University, Wuhan, China; ^3^Key Laboratory of Development of Veterinary Diagnostic Products, Ministry of Agriculture, College of Veterinary Medicine, Huazhong Agricultural University, Wuhan, China; ^4^The Cooperative Innovation Center for Sustainable Pig Production, Huazhong Agricultural University, Wuhan, China

**Keywords:** polymerase, pdm/09, innate immunity, signaling pathway, phosphorylation

## Abstract

The influenza A virus (IAV) can be recognized by retinoic acid-inducible gene I (RIG-I) to activate the type I interferon response and induce antiviral effects. The virus has evolved several strategies to evade the innate immune response, including non-structural protein 1 (NS1) and its polymerase subunits. The mechanism by which NS1 inhibits interferon-β (IFN-β) is well understood, whereas the mechanism by which polymerase acid protein (PA) inhibits IFN-β remains to be elucidated. In this study, we observed that the IAV PA protein could inhibit the production of IFN-β and interferon-stimulated genes induced by Sendai virus through interferon regulatory factor 3 (IRF3), but not through nuclear factor-kappaB (NF-kappaB). In addition, PA inhibited IFN-β induction by RIG-I, melanoma differentiation-associated gene 5, mitochondria antiviral signaling protein, TANK-binding kinase 1, inhibitor of nuclear factor kappa-B kinase-ε (IKKε), and IRF3 overexpression. Furthermore, PA interacted with IRF3 to block its activation. The N-terminal endonuclease activity of PA was responsible for its interaction with IRF3 and inhibition of the IFN-β signaling pathway. In summary, our data reveal the mechanism by which IAV PA inhibits the IFN-β signaling pathway, providing a new mechanism by which the virus antagonizes the antiviral signaling pathway.

## Introduction

Type I interferon (IFN-I) is the first defense line of the host antiviral response and leads to broad-spectrum antiviral effects (1–4). Upon Influenza A virus (IAV) infection, viral RNA (vRNA) is recognized by pathogen recognition receptors (PRRs) and initiates the innate immune response. The activation of innate immunity leads to a cascade of downstream signaling pathways and results in the activation of IFN-I and a variety of inflammatory cytokines (5, 6). Both retinoic acid-inducible gene 1 (RIG-I) and melanoma differentiation-associated gene 5 (MDA-5) (5, 7–9) can recognize IAV RNA and bind to the mitochondria antiviral signaling protein (MAVS, also known as IPS-1), thereby activating TANK-binding kinase 1 (TBK-1) and inhibitor of nuclear factor kappa-B kinase (IKK). These recruits could lead to the activation of interferon regulatory factor 3 (IRF3) and nuclear factor-kappaB (NF-kappaB) (1, 10). Activation of IRF3 depends on the phosphorylation of five serine residues at its C-terminus, leading to its dimerization and nuclear localization (11) and initiating the transcription of IFN and IFN-induced genes (12, 13).

During IAV infection, polymerase acid protein (PA), PB1, and PB2, three components of the vRNA polymerase, are responsible for viral replication and transcription (14–17). To evade the antiviral response of the host immunity, IAV has developed a series of mechanisms to antagonize IFNs. It has been reported that the IAV polymerases could inhibit interferon-β (IFN-β) expression (18–20). PB2 and PB1 could interact with MAVS and independently inhibit IFN-β promoter activation (21). PB2-588I could exacerbate PB2 inhibition of IFN-β and contribute to high virulence (22). In addition, the non-structural protein 1 (NS1) binds to tripartite motif containing 25 (TRIM25) and blocks the recognition of host RIG-I, inhibiting IFN-β induction (23).

The PA subunit has been reported to enhance influenza virus polymerase activity, pathogenicity, transmission, contribute to its capacity to infect a range of hosts (24–26), and has also been suggested to be involved in the inhibition of IFN-β induction (21); however, this mechanism still remains to be elucidated. In this study, we demonstrated that IAV PA could block IRF3 activation and suppress IFN-β production. The aspartic acid at position 108 in PA is required for this activity.

## Materials and Methods

### Cell Culture

Human embryonic kidney (293T) cells were maintained in RMPI 1640 (Invitrogen, Carlsbad, CA, USA) with 10% fetal bovine serum (FBS). Human type 2 alveolar epithelial (A549) cells were maintained in F12 (Invitrogen) with 10% FBS.

### Plasmid Construction

The IFN-β-luc, IRF3-luc, NF-kappaB-luc, RL-TK, RIG-I, MDA-5, MAVS, TBK-1, IKKε, TNF receptor-associated factor 3 (TRAF3), and IRF3 plasmids were kindly provided by Zhengfan Jiang (Peking University) (22). The PA expression plasmids were generated from A/Mexico/4486/2009 (H1N1) (pdm/09 PA), A/swine/Nanchang/F9/2010 (H1N1) (F9), A/duck/Hubei/Hangmei01/2006 (H5N1), and A/Shanghai/02/2013 (H7N9) and were cloned into the p3xFlag-CMV-14 vector. The N-terminal PA fragment (PAN), C-terminal PA fragment (PAC), and PA-D108A mutants and IRF3 were cloned into both the p3xFlag-CMV-14 and pCAGGS-HA vectors.

### Luciferase Assay

To determine the impact of PA on the promoter activities of IFN-β, IRF3, or NF-kappaB, the luciferase reporter plasmids were co-transfected with the indicated plasmids in 293T cells. A co-transfected pRL-TK vector that provided constitutive expression of Renilla luciferase was used as a control (Promega, Madison, WI, USA). After 24 h, cells were infected with 10 hemagglutinating activity units/well of Sendai virus (SEV; Centre of Virus Resource and Information, Wuhan Institute of Virology, Chinese Academy of Sciences) or they were transfected with 200 ng Poly(I:C) (Sigma, St. Louis, MO, USA) for 8 h. Cells were then lysed for measuring the luciferase activity using the dual-luciferase assay system (Promega) according to the manufacturer’s instructions.

### RNA Extraction and Real-time q-PCR

A549 cells in 6-well plates were transfected with empty vector or a plasmid encoding pdm/09 PA, pdm/09 PAN, pdm/09 PAC, or pdm/09 PA-D108A mutant protein at the indicated quantities. After 24 h, the cells were infected with SEV for 8 h or left uninfected. Total RNA was extracted from A549 cells using TRIzol (Invitrogen), and 1 µg of RNA was treated with DNase (Promega). The cDNA was treated with avian myeloblastosis virus (AMV) reverse transcriptase and an oligo(dT-)18-adaptor primer (TaKaRa Biotechnology, DaLian, China). The reaction mixtures were incubated at 42°C for 1 h and were terminated by heating at 95°C for 5 min. The primers used in real-time q-PCR were as follows: IFN-β, 5′-TCTTTCCATGAGCTACAACTTGCT-3′ (forward), 5′-GCAGTATTCAAGCCTCCCATTC-3′ (reverse); interferon-stimulated gene (ISG)-15, 5′-CGCAGATCACCCAGAAGATCG-3′ (forward), 5′-TTCGTCGCATTTGTCCACCA-3′ (reverse); ISG-56, 5′-GCTTTCAAATCCCTTCCGCTAT-3′ (forward), 5′-GCCTTGGCCCGTTCATAAT-3′ (reverse); C-X-C motif chemokine (CXCL) 10, 5′-GTGGCATTCAAGGAGTACCTC-3′ (forward), 5′-TGATGGCCTTCGATTCTGGATT-3′ (reverse); and GAPDH, 5′-TCATGACCACAGTCCATGCC-3′ (forward), 5′-GGATGACCTTGCCCACAGCC-3′ (reverse) (22). The assay was performed on an ABIViiA 7 PCR system (Applied Biosystems, Waltham, MA, USA) in a total volume of 10 µl per sample, containing 5 µl of 2× SYBR Green Master Mix (Roche, Indianapolis, IN, USA), 0.5 µl of cDNA, 0.25 µl of each primer (10 mM), and 4 µl of DEPC-treated water. The transcript level of each gene was normalized using GAPDH as a control.

### ELISA of IFN-β

To measure IFN-β secretion, A549 cells were transfected with an empty vector or plasmids with Flag-tagged pdm/09 PA. After 24 h, the cells were left uninfected or were infected with SEV for 8 h. The supernatants were harvested for an ELISA using a human IFN-β ELISA kit (Elabscience, Wuhan, China) according to the manufacturer’s instructions.

### Co-Immunoprecipitation (CO-IP) and Western Blotting

293T cells cultured in 6-well plates were transfected with the indicated plasmids. After 24 h, the cells were lysed and analyzed by SDS-PAGE or in NP-40 buffer (SDS free) for NATIVE-PAGE. The Western blotting was performed using monoclonal anti-Flag antibody (Sigma), monoclonal anti-HA antibody (ABclonal, Cambridge, MA, USA), an IRF3 polyclonal antibody (Proteintech, Rosemont, IL, USA), anti-phospho IRF3 (ser386) antibody (Merck Millipore, Darmstadt, Germany), IAV PA polyclonal antibody (Gene Tex, Irvine, CA, USA), anti-NF-kappaB antibody(Cell Signaling Technology, 3 Trask Lane, Danvers, MA, USA), or anti-phospho NF-kappaB antibody(Cell Signaling Technology). For CO-IP, the 293T cells were plated in a 15-cm dish with 70% cells and then transfected with 10 µg of plasmid. After 24 h, the cells were collected, washed in ice-cold PBS three times, and lysed in 1 ml of Tris lysis buffer (Cell Signaling Technology) on ice for at least 20 min. The supernatant (400 µl) was used for immunoprecipitation with 5 µl of monoclonal anti-Flag antibody (Sigma) or 5 µl of normal rabbit IgG control (ABclonal). The cell lysates and immunoprecipitates (IPs) were analyzed by SDS-PAGE.

### Cytokine Treatment

293T cells were transfected with indicated quantities of Flag-tagged pdm/09 PA. After 24 h, cells were treated with 100 U/mL of human IFN-β (R&D Systems, Minneapolis, MN, USA) for 1 h. Cells were then lysed for Western blot using anti-STAT1 (9H2) antibody (Cell Signaling Technology) and anti-phospho STAT1 (Tyr701) antibody (Cell Signaling Technology).

### Indirect Immunofluorescence Assay

293T cells seeded onto coverslips and placed into 12-well plates were transfected with the indicated plasmids or an empty vector and cultured in the presence or absence of SEV for 8 h. The cells were fixed with 4% paraformaldehyde for at least 15 min, treated with 0.2% Triton X-100 for 15 min, and blocked with bovine serum albumin (5%) in PBS for 2 h at room temperature. The cells were then incubated separately with monoclonal anti-Flag antibody (Sigma) or IRF3 polyclonal antibody (Proteintech) at a dilution of 1:200, followed by incubation with a fluorescein isothiocyanate-labeled secondary antibody (Invitrogen) for 1 h at 37°C. Nuclei were stained with 4′,6-diamidino-2-phenylindole (DAPI) for 15 min. The images were taken using a Zeiss LSM510 Meta confocal microscope (Carl Zeiss, Zena, Germany).

### Statistical Analyses

Statistical analyses for all experiments were performed using Student’s *t*-test or one-way ANOVA (for more than two groups) with GraphPad Prism software (San Diego, CA, USA). The data were representative of at least three separate experiments. The error bars represent the SDs, and *p* values <0.05 are significant (*p < 0.05, **p < 0.01, ***p < 0.001).

## Results

### PA Antagonizes SEV-Induced IFN-β Production

**Figure 1:**
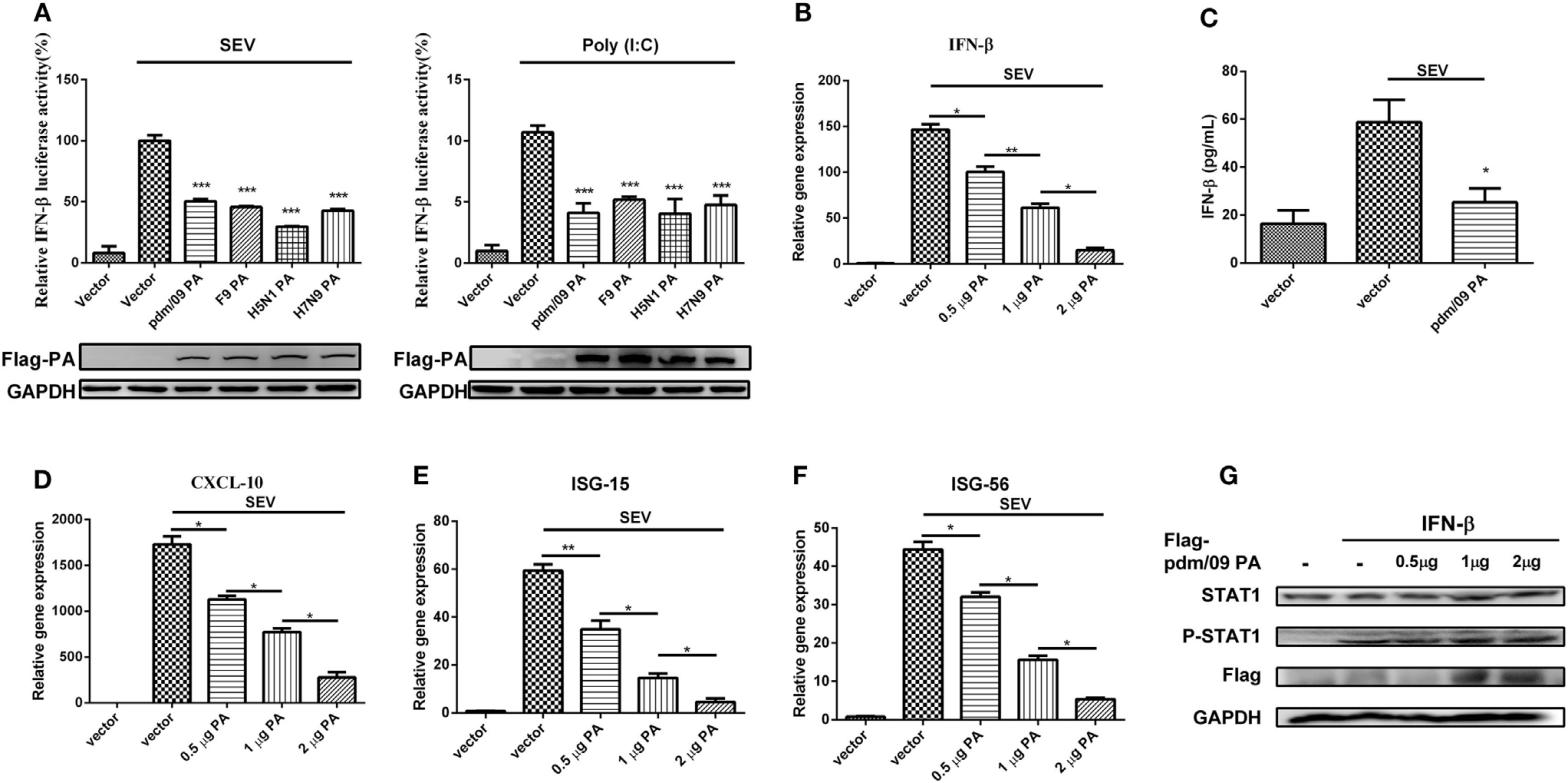
Inhibition of SEV-induced interferon-β (IFN-β) expression by PA. 293T cells in 12-well plates were transfected with 0.5 µg of Flag-tagged pdm/09 PA, F9PA, H5N1PA, H7N9PA, or an empty vector, together with 0.3 µg of IFN-β-luc and 0.02 µg of RL-TK. After 24 h, cells were infected with SEV or transfected with 200 ng of Poly(I:C) for 8 h and lysed for luciferase assay **(A)**. A549 cells in 6-well plates were transfected with indicated quantities of Flag-tagged pdm/09 PA or empty vector. After 24 h, cells were infected with SEV or left uninfected for 8 h. The supernatants were harvested for IFN-β ELISA **(C)**. The cells were harvested, and total RNAs were extracted for detecting the expression levels of IFN-β **(B)**, CXCL-10 **(D)**, ISG-15 **(E)**, and ISG-56 **(F)** by real-time q-PCR. 293T cells were transfected with increasing quantities of Flag-tagged pdm/09 PA for 24 h and then treated with human IFN-β for 1 h or left untreated. Then, cells were lysed for Western blot using anti-STAT1 and anti-phospho STAT1 antibodies **(G)**. The bars represent the SEs of the means, based on three experiments. *p < 0.05, **p < 0.01, ***p < 0.001 [as determined by Student’s *t*-test **(A,C)** or by one-way ANOVA **(B,D–F)**].

To investigate the regulation of influenza virus PA in IFN-β induction, we constructed the expression plasmid of influenza A (H1N1) pdm09 virus (pdm/09) PA and a series of influenza PA expression plasmids from different influenza virus subtypes, including F9PA, H5N1PA, and H7N9PA. Luciferase assays revealed that all subtypes of PA could strongly inhibit SEV- and Poly(I:C)-induced IFN-β promoter activity (Figure 1A), supporting the hypothesis that IAV PA plays a role in inhibition of the IFN-β signaling pathway (21). This hypothesis was further supported by the fact that IFN-β induction by SEV could be further inhibited by pdm/09 PA overexpression, as evidenced by reduced IFN-β transcription and protein levels (Figures 1B,C). Because of the inhibition of the IFN-β signaling pathway, CXCL-10 (also known as IP-10) (Figure 1D), ISG-15 (Figure 1E), and ISG-56 (Figure 1F) induction levels were all strongly reduced by pdm/09 PA. Furthermore, pdm/09 PA expression showed no influence on IFN-β-induced phosphorylation of signal transducer and activator of transcription 1 (STAT1) (Figure 1G). Therefore, this study indicates that influenza PA can antagonize IFN-β induction.

### Pdm/09 PA Inhibits the RIG-I-Like Receptor (RLR)-Mediated IFN-β Signaling Pathway through IRF3, rather than NF-kappaB

**Figure 2:**
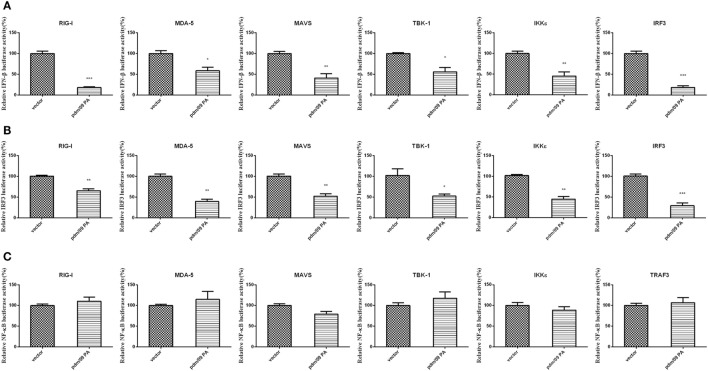
Inhibition of RIG-I-like receptor (RLR)-mediated IFN-β signaling pathway by Pdm/09 PA 293T cells in 12-well plates were transfected with 0.5 µg of Flag-tagged pdm/09 PA or empty vector, together with 0.3 µg of IFN-β-luc **(A)** or 0.3 µg of IRF3-luc **(B)** or 0.3 µg of NF-kappaB-luc **(C)**, 0.02 µg of RL-TK, and 0.5 µg of transcription factors as the positive controls. After 24 h, the cells were lysed for luciferase assay. The bars represent the SEs of the means, based on three experiments. *p < 0.05, **p < 0.01, ***p < 0.001 (as determined by Student’s *t*-test).

During influenza virus infection, vRNAs will be recognized by PRRs and trigger the activation of transcription factors, leading to IFN-β induction (10). To describe the molecular mechanism of IFN-β inhibition by PA, a luciferase assay of the IFN-β promoter was performed with several activators, including RIG-I, MDA-5, MAVS, TBK-1, IKKε, and IRF3. PA induced by upstream factors could inhibit IFN-β promoter activation (Figure 2A). The assay indicated that PA may inhibit the IFN-β pathway downstream of these activators.

Because activation of the transcription factors IRF3 and NF-kappaB is essential for IFN-β production, we further investigated whether pdm/09 PA disturbs the IRF3 or NF-kappaB pathway using luciferase assays of the IRF3 (Figure 2B) or NF-kappaB promoter (Figure 2C). Interestingly, IRF3 promoter activity induced by the above activators was strongly inhibited. However, NF-kappaB promoter activity was not inhibited by pdm/09 PA protein. Therefore, we conclude that PA can interfere in RLR-mediated IFN-β production through the IRF3-dependent signaling pathway.

### Pdm/09 PA Interacts with IRF3 and Suppresses IRF3 Phosphorylation, Dimerization, and Subsequent Nuclear Translocation

**Figure 3:**
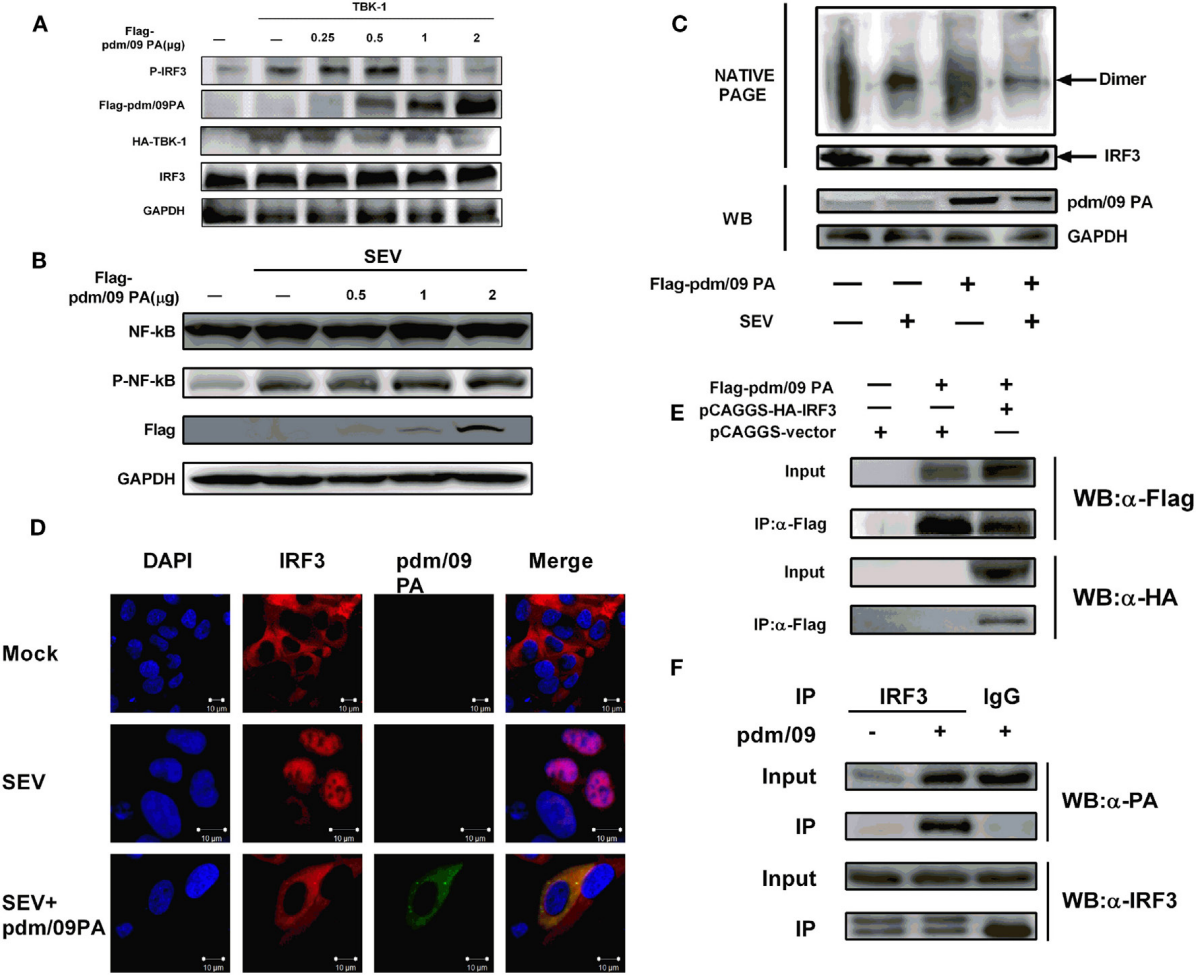
Inhibition of interferon regulatory factor 3 (IRF3) activation is mediated by interaction between Pdm/09 polymerase acid protein (PA) and IRF3 **(A)** 293T cells in 6-well plates were transfected with increasing quantities of Flag-tagged pdm/09 PA. After 24 h, cells were lysed, and the expression levels of IRF3, P-IRF3, and GAPDH were measured with indicated antibodies by Western blotting. **(B)** 293T cells in 6-well plates were transfected with increasing quantities of Flag-tagged pdm/09 PA. After 24 h, cells were infected with SEV or left uninfected for 8 h. Then the cells were lysed for Western blotting using nuclear factor-kappaB (NK-kappaB) and phospho-NF-kappaB antibodies. **(C)** 293T cells in 6-well plates were transfected with Flag-tagged pdm/09 PA or empty vector. After 24 h, cells were infected with Sendai virus (SEV) or were left uninfected for 8 h and then they were harvested for NATIVE-PAGE using anti-IRF3 antibody or for Western blot using anti-Flag and anti-GAPDH antibodies. **(D)** 293T cells seeded onto coverslips and placed into 12-well plates were transfected with Flag-tagged pdm/09 PA or empty vector. After 24 h, cells were infected with SEV or were left uninfected for 8 h and were fixed for immunofluorescence assay (IFA), with endogenous IRF3 (red), PA (green), and nuclei (blue) labeled with anti-IRF3 antibody, anti-Flag antibody and DAPI, respectively, using confocal microscopy. **(E)** 293T cells in 6-well plates were transfected with Flag-tagged PA and HA-tagged IRF3. After 24 h, cells were lysed and precipitated with anti-Flag antibody. The cell lysates and immunoprecipitates (IPs) were analyzed by Western blotting using anti-Flag and anti-HA antibodies. **(F)** 293T cells were infected with H1N1 pdm/09 ([MOI] = 0.1) for 12 h, and cells were then lysed and precipitated with anti-IRF3 antibody or with normal IgG as a negative control. The cell lysates and IPs were analyzed by Western blotting using anti-IRF3 and anti-PA antibodies.

In the context of viral infection, activation of signaling pathways can induce IRF3 phosphorylation and dimerization, which results in IRF3 accumulation in the nucleus (11, 27). We observed that pdm/09 PA overexpression could decrease IRF3 phosphorylation in a dose-dependent manner but could not influence NF-kappaB activation (Figures 3A,B). In addition, pdm/09 PA could also suppress SEV-induced dimerization (Figure 3C) and nuclear translocation of IRF3 (Figure 3D). These data indicated that the IAV PA protein inhibits IRF3 activation in response to SEV stimulation. Interestingly, pdm/09 PA and IRF3 showed co-localization in the cytoplasm by confocal microscopy (Figure 3D).

Since pdm/09 PA antagonizes IRF3 activation, we hypothesized that both proteins interact. A CO-IP experiment showed that pdm/09 PA does co-precipitate with IRF3 in both transfected cells (Figure 3E) and virus-infected cells (Figure 3F). These data indicate that pdm/09 PA interacts with IRF3 and further blocks IRF3 activation.

### The N-Terminal Functional Domain of pdm/09 PA Is Responsible for IFN-β Suppression

**Figure 4:**
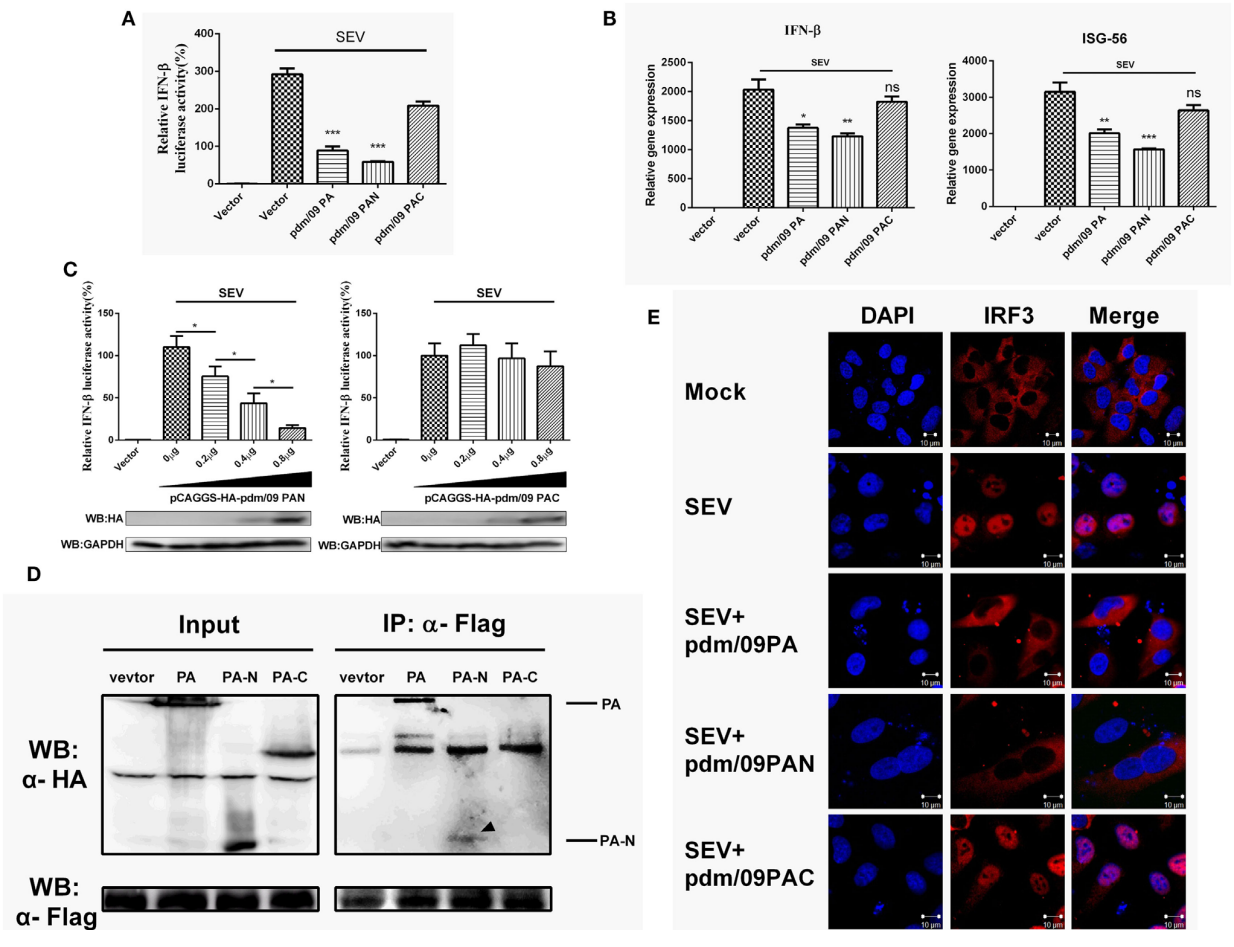
Interferon-β (IFN-β) induction was suppressed by the N-terminus of pdm/09 polymerase acid protein (PA). **(A)** 293T cells in 12-well plates were transfected with 0.5 µg of Flag-tagged pdm/09 PA, pdm/09 N-terminal PA fragment (PAN), pdm/09 C-terminal PA fragment (PAC), or an empty vector, together with 0.3 µg of IFN-β-luc and 0.02 µg of RL-TK. After 24 h, cells were infected with SEV or were left uninfected for 8 h and then they were lysed for the luciferase assay. **(B)** A549 cells in 6-well plates were transfected with 1 µg of Flag-tagged pdm/09 PA, pdm/09 PAN, pdm/09 PAC, or empty vector. After 24 h, cells were infected with SEV for 8 h and subsequently harvested, and total RNA was then extracted for detection of IFN-β and ISG-56 expression levels by real-time q-PCR. **(C)** 293T cells in 12-well plates were transfected with HA-tagged PAN and PAC in increasing quantities and co-transfected with 0.3 µg of IFN-β-luc and 0.02 µg of RL-TK. After 24 h, cells were infected with SEV for 8 h and then lysed for use in the luciferase assay. **(D)** 293T cells in 6-well plates were transfected with 2 µg of HA-tagged pdm/09 PA, pdm/09 PAN, pdm/09 PAC, or an empty vector, and Flag-tagged IRF3. After 24 h, cells were lysed and precipitated with anti-Flag antibody. The cell lysates and IPs were analyzed by Western blotting using anti-Flag and anti-HA antibodies. **(E)** 293T cells were seeded onto coverslips and placed into 12-well plates and were transfected with Flag-tagged pdm/09 PA, pdm/09 PAN, pdm/09 PAC, or empty vector. After 24 h, cells were infected with SEV or were left uninfected for 8 h and were fixed for IFA, with endogenous IRF3 (red) and nuclei (blue) shown with anti-IRF3 antibody and DAPI via confocal microscopy. The bars represent the SEs of the means, based on three experiments. *p < 0.05, **p < 0.01, ***p < 0.001 [as determined by Student’s *t*-test **(A,B)** or by one-way ANOVA **(C)**].

Influenza A virus (IAV) PA consists of two functional domains: a 30-kDa N-terminal fragment (PAN, residues 1–257) and a 53-kDa C-terminal fragment (PAC, residues 258–716) (28). PAC is known as the PB1 binding domain, while PAN possesses endonuclease activity (29). Similar to what was observed for full-length PA, pdm/09 PAN could strongly inhibit SEV-induced IFN-β promoter activation in a dose-dependent manner (Figures 4A,C). Furthermore, the expression levels of IFN-β and ISG-56 were reduced by pdm/09 PA and pdm/09 PAN (Figure 4B), whereas pdm/09 PAC showed no significant effect on IFN-β induction (Figure 4C). These results indicate that the N-terminus of pdm/09 PA may be responsible for IFN-β suppression.

Because PA interacts with IRF3 and blocks its activation (Figure 3), we further investigated the interaction of PAN with IRF3. Similar to what was observed with pdm/09 PA, PAN was shown to interact with IRF3 in a CO-IP assay (Figure 4D) and to block SEV-induced translocation of IRF3 into the nucleus (Figure 4E). Therefore, the N-terminus of pdm/09 PA is responsible for the interaction with IRF3 and suppression of the IFN-β signaling pathway.

### The Binding Activity of pdm/09 PA to IRF3 Is Dependent on Asp108

**Figure 5:**
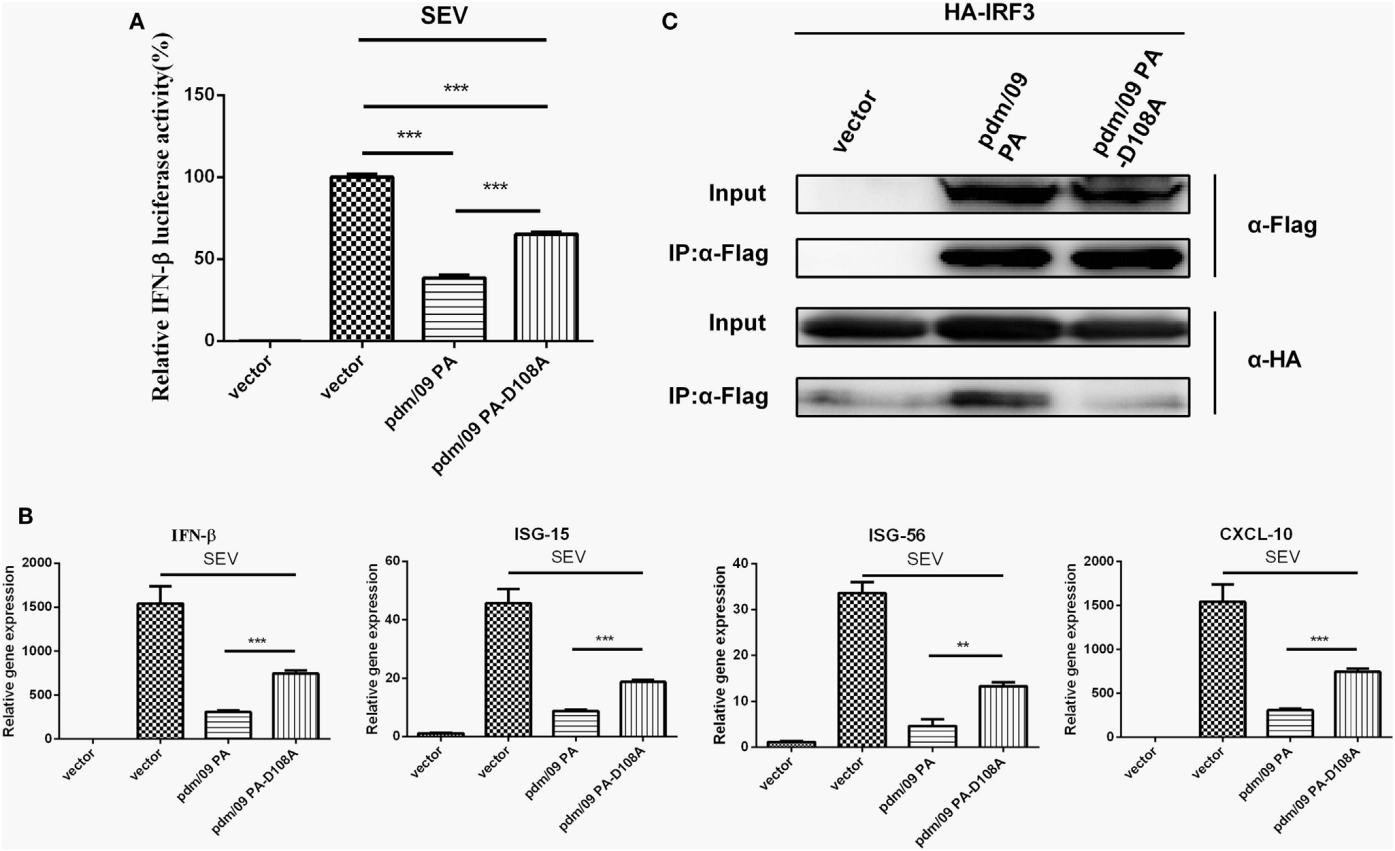
The binding activity of pdm/09 polymerase acid protein (PA) to interferon regulatory factor 3 (IRF3) is dependent on Asp108. **(A)** 293T cells in 12-well plates were transfected with 0.5 µg of Flag-tagged pdm/09 PA, pdm/09 PA-D108A mutant or empty vector, together with 0.3 µg of interferon-β (IFN-β)-luc and 0.02 µg of RL-TK. After 24 h, cells were infected with Sendai virus (SEV) or were left uninfected for 8 h and were then lysed for the luciferase assay. **(B)** A549 cells in 6-well plates were transfected with 2 µg of Flag-tagged pdm/09 PA, pdm/09 PA-D108A mutant or an empty vector. After 24 h, cells were infected with SEV or were left uninfected for 8 h. The cells were harvested, and total RNA was extracted for detection of IFN-β, CXCL-10, ISG-15, and ISG-56 expression levels by real-time q-PCR. **(C)** 293T cells in 6-well plates were transfected with 2 µg of Flag-tagged pdm/09 PA, pdm/09 PA-D108A mutant or an empty vector, and HA-tagged IRF3. After 24 h, cells were lysed and precipitated with anti-Flag antibody. The cell lysates and immunoprecipitates (IPs) were analyzed by Western blotting using anti-Flag and anti-HA antibodies. The bars represent the SEs of the means, based on three experiments. *p < 0.05, **p < 0.01, ***p < 0.001 (as determined by Student’s *t*-test).

Because the N-terminus of pdm/09 PA contains an endonuclease domain, it is important to investigate the role that the endonuclease activity of pdm/09 PA plays in inhibition of the host IFN-β induction. A previous study showed that a PA-D108A mutant could abolish the endonuclease activity of PA *in vitro* (30). The D108A mutation impairs the capacity of pdm/09 PA to inhibit IFN-β induction by SEV at the transcriptional (Figure 5A) and translational levels (Figure 5B). In addition, ISG-15, ISG-56, and CXCL-10 expression levels in cells transfected with the pdm/09 PA-D108A mutant were only half that of those in cells transfected with pdm/09 PA (Figure 5B). Furthermore, the mutation abolished the interaction between IRF3 and pdm/09 PA (Figure 5C). Together, these results indicate that Asp108 of pdm/09 PA contributes to the interaction of pdm/09 PA with IRF3 and inhibition of the IFN-β signaling pathway.

## Discussion

The influenza virus RNA polymerase is a heterotrimeric complex consisting of the PB2, PB1, and PA subunits, all of which are required for vRNA transcription and replication (15–17). Infection is usually associated with inhibition of the host antiviral response by viral polymerases (31). Upon infection, IAV triggers the activation of the host innate immunity (32), which leads to IFN-β secretion, which mediates an antiviral effect. Hence, IAV employs a variety of strategies to circumvent innate immunity and the host’s IFN response. The influenza viral NS1 protein and polymerase proteins could reduce IFN-β synthesis through the inhibition of IFN signaling pathways, thereby circumventing the antiviral effect of host immunity, which is essential for IAV infection (18, 19, 21, 23, 33–36). However, the mechanism by which PA inhibits IFN-β remains unknown. In our study, we found that all the PA subunits from the IAV strains H1N1, H5N1, and H7N9 could antagonize IFN-β production through IRF3 rather than NF-kappaB.

It is known that upon viral infection, IRF3 is phosphorylated, exposing the IRF association domain at the C-terminus. Subsequently, phosphorylated IRF3 will be dimerized and translocate to the nucleus (37), leading to IFN-β transcription (11, 27, 38–41). In this study, we observed that IRF3 phosphorylation and dimerization were inhibited by pdm/09 PA, resulting in IRF3 accumulation in the cytoplasm. Interestingly, IRF3 and pdm/09 PA were co-localized, and a CO-IP assay further indicated an interaction between pdm/09 PA and IRF3. These results explain the findings that PA can antagonize IFN-β production through IRF3.

The influenza viral PA protein can be digested by trypsin through two domains: the N-terminal domain (from amino acid residues 1–257) and the C-terminal domain (from amino acid residues 277–716). Amino acid residues 257–276 provide a flexible structure to ensure the connection of PA to PB1 (28, 30). The N-terminus of PA possesses pleiotropic functions (42), including the host RNA cap-snatching via an endonuclease activity (15, 43, 44) and proteolysis induction (45, 46). Both functions may cause non-specific reductions in host gene expression levels. The capacity of PA to inhibit the IRF-3 signaling pathway, but not the NF-kappaB signaling pathway, suggests that PA inhibits IFN-β via some specific molecular mechanism. To further investigate the precise function of the domains of PA against the host IFN response, we generated plasmids pdm/09 PAN (1–257) and pdm/09 PAC (258–716) and found that PAN activity was similar to that of full-length PA and that pdm/09 PAN could interact with IRF3 and effectively alter the distribution of endogenous IRF3 upon SEV infection.

Furthermore, mutation of PA (PA-D108A mutant) causes loss of endonuclease activity (30), abolishes the interaction of PA with IRF3, and decreases the inhibition of the IFN-β signaling pathway. These data further support our opinion that the N-terminal endonuclease activity may be required for PA to interact with IRF3 and then block its activation.

Regrettably, we failed to generate a recombinant virus carrying the pdm/09 PA-D108A mutation to evaluate the effect of IFN-β inhibition by PA on virulence. The inability to generate the recombinant virus may be due to the essential function of PA endonuclease activity for viral propagation, such as viral genes transcription and the vRNA promoter binding activity (30). Considering that the sequence of the endonuclease domain of PA is highly conserved, we hypothesized that IAV PA could bind to IRF3 through its N-terminal domain and suppress IFN-β induction, although the exact pathogenic role of PA during infection was difficult to be elucidated.

It has been reported that a single amino acid substitution of PB2 (N9D) blocks mitochondrial localization of the PB2 protein and suppresses IFN-β induction (18), while we demonstrated that PA blocks IRF3 activation and IFN-β induction dependent on Asp108. In addition, PA could mediate IFN-β induction through multiple mechanisms (as shown in Figure 5, the pdm/09 PA-D108A mutant is still able to inhibit host IFN-β induction). It is likely that the PA protein impacts the synthesis of host proteins through its helix α4 domain and amino residues 51–57, the flexible loop domain (43, 47). The first 85 amino acid residues of PA and amino acid residues from 186 to 247, which are necessary to induce proteolysis of PA, may also contribute to suppression of host IFN-β induction (45). There may also be other mechanisms by which PA inhibits IFN-β that remain unknown.

In summary, we observed that IAV PA can interact with IRF3 and inhibit its activation, thereby blocking the activation of the IFN-β signaling pathway. The interaction requires the N-terminal endonuclease activity of PA. This study reveals a new mechanism by which IAV inhibits the IFN-β signaling pathway.

## Author Contributions

CY, ZZ, AZ, and MJ designed the study; CY and ZZ performed experiments; CY, ZZ, SW, XS, and DZ analyzed the data; CY drafted the manuscript; XMS, AZ, and MJ contributed to conduct of the laboratory work and critical review of the manuscript. All authors contributed to read and approved the final version.

## Conflict of Interest Statement

The authors declare that the research was conducted in the absence of any commercial or financial relationships that could be construed as a potential conflict of interest.
